# Distribution of Adiponectin Receptors 1 and 2 in the Rat Olfactory Bulb and the Effect of Adiponectin Injection on Insulin Receptor Expression

**DOI:** 10.1155/2017/4892609

**Published:** 2017-12-31

**Authors:** Alfredo Miranda-Martínez, Octavio Fabián Mercado-Gómez, Virginia Arriaga-Ávila, Rosalinda Guevara-Guzmán

**Affiliations:** ^1^Departamento de Fisiología, Facultad de Medicina, Universidad Nacional Autónoma de México, Del. Coyoacán, 04510 Ciudad de México, Mexico; ^2^Programa de Doctorado en Ciencias Biológicas, Coordinación del Posgrado en Ciencias Biológicas, Edificio B, 1° Piso. Circuito de Posgrados, Ciudad Universitaria, Del. Coyoacán, 04510 Ciudad de México, Mexico

## Abstract

**Background:**

Adiponectin (APN) is an adipocyte-derived hormone that has peripheral beneficial effects. Although its receptors AdipoR1 and AdipoR2 are expressed in the brain, their function in neurons is poorly understood. The aims of this work were to describe the distribution of APN receptors in the olfactory bulb (OB) as well as the possible effects of APN injection on the insulin receptor (InsR) content and Akt kinase.

**Method:**

We performed the double immunofluorescence technique to describe the distribution of AdipoRs and the cellular type they were expressing. mRNA transcript and protein content were assessed by RT-PCR and Western blot, respectively. APN injection was performed to analyze its possible effect on the insulin pathway.

**Results:**

We found that AdipoRs were localized in all cell layers and in both neurons and astrocytes. We observed the presence of mRNA transcripts and immunoblot analysis confirmed the protein on the intact OB; APN injection in the OB resulted in a slight decrease of the total InsR and Akt phosphorylation and a reduction of phopho-InsR content.

**Conclusions:**

These data demonstrated that AdipoRs are expressed in OB regions, and APN injection could act as an insulin pathway modulator in the OB and thus possibly contribute to olfaction physiology.

## 1. Introduction

Research efforts have demonstrated that the central nervous system (CNS) is tightly linked with the endocrine system to regulate the food intake and energy balance. Thus, a variety of hormones are released by the gastrointestinal tract, associated glands, or peripheral tissues which are carried by the bloodstream to exert their functions in distant targets [1].

Adiponectin (APN) is a hormone mainly secreted from adipocytes [2] that has been involved in several physiological functions. Specifically, APN is associated with improving insulin sensitivity, glucose uptake, and lipid metabolism [3–5]; as modulator of the endothelial function or exerting antiatherogenic, anti-inflammatory, and cardioprotective effects [6, 7]. Interestingly, the low plasma level of APN contributes to the development of metabolic and cardiovascular diseases [8–10] and results in a risk factor for neurodegenerative disorders, including Alzheimer's disease [11–13].

Previous studies indicate that adiponectin receptors 1 and 2 (AdipoR1 and AdipoR2) are widely expressed throughout the central nervous system (CNS), including cortex, hypothalamus, and hippocampus [14]. It is well known that APN acts centrally to control energy metabolism [15] and also as an important neuroprotective hormone [16–19] because of its neurotrophic factor activity which benefits neural function [20].

On the other hand, the olfactory system is essential for the survival of many animal species, providing sensorial information about food and environment, as well as influencing social and sexual behaviors [21]. A physiological role of APN over the olfactory system, especially olfactory bulbs (OB), has not been investigated in detail. Nevertheless, Hass and colleagues have shown the presence of AdipoR1 transcript in the mouse olfactory mucosa and postulated its possible role in the nutritional status of the body [22]. Furthermore, it was recently demonstrated that APN increases the amplitude of response in olfactory epithelial cells under odor stimulation indicating that APN acts as an orexigenic signal that it can modulate the response of the olfactory neurons independently of which odorant receptors are being expressed [23].

It is well known that the brain is an insulin-sensitive organ and the presence of insulin and its specific receptor in several brain regions has been well described [24]. In this regard, the insulin receptor (InsR) is highly expressed in the hypothalamus, the hippocampus, and the OB, brain-related structures implicated in glucoregulation, feeding regulation, circuit development, food intake, and cognitive processing [24–26]. Interestingly, APN is described to act primarily as an insulin-sensitizing hormone, being the liver and the skeletal muscle its main targets; however, it is not well known to what level APN is exerting its insulin sensitivity effect [14]. In this regard, we hypothesize that APN could be acting as a modulator of InsR activity in different regions of the rat's brain, particularly OB, similarly to occur in peripheral tissues. Therefore, the aims of this study were to describe the punctual presence and distribution of AdipoR1 and AdipoR2 in the OB of Wistar male rats and to investigate the effects of APN administration on InsR content and insulin signaling in this brain region.

## 2. Materials and Methods

### 2.1. Animals

This study was conducted in accordance with the guidelines and requirements of the World Medical Association Declaration of Helsinki (1964) and approved by the Ethics Committee of the Medicine School at the Universidad Nacional Autónoma de Mexico (UNAM), number FISRGG02021 and regulated by the Mexican Official Norm NOM-062-ZOO-1999 to minimize animal suffering. Adult male Wistar rats (250–300 g) were used and maintained under a 12 h light-dark cycle with free access to water and standard laboratory chow (PMI Nutrition International Inc., Greenwood, MO). Treatments were always conducted in the morning (10 : 00 am) to avoid circadian effects, and the results show a comparison between vehicle-injected versus ADP-injected animals.

### 2.2. Immunofluorescence for AdipoR1 and AdipoR2

Animals (*n* = 6, intact animals) were deeply anesthetized under sodium pentobarbital overdose (100 mg/kg i.p.) and then transcardially perfused with 200 ml of phosphate buffered saline (PBS) solution (pH = 7.4, 4°C) followed by 4% paraformaldehyde in phosphate buffer (0.1 M, pH 7.4). The OBs were dissected and dehydrated in alcohol solutions (100 to 70%), xilol solution, and mounted in paraffin wax. Coronal sections of 5 *μ*m thickness of the OB were obtained using a microtome (Leica Biosystems, Germany) and mounted on poly-L-lysine-treated glass slides. To reduce autofluorescence of the OB sections, we submerge the slides into a Coplin glass filled with a saturated solution of Sudan black B (0.25%) in 70% isopropyl alcohol for 90 min. Then, slides were rinsed with 70% isopropyl alcohol and distillated water and immediately after, OB sections were rinsed with PBS for 5 min and blocked with 1% bovine serum albumin (BSA) in PBS solution for 90 min. Then, antibodies against both adiponectin receptors AdipoR1 (anti-goat antibody, 1 : 250, Santa Cruz Biotechnology, sc-46748, USA); AdipoR2 (anti-rabbit antibody 1 : 200, Santa Cruz Biotechnology, sc-99184, USA); the neuronal marker NeuN (anti-mouse antibody clone A60 1 : 100, Millipore MAB377, USA) and the astrocyte marker GFAP (anti-mouse antibody 1 : 100, Biocare, USA) were incubated together in blocking solution at 4°C overnight. Anti-mouse, anti-goat, and anti-rabbit 488 and 594 Alexa Fluor dye coupled secondary antibodies (1 : 500, Thermo Fisher Scientific, USA) were also incubated together in blocking solution during 1 h in room temperature, and then slides were rinsed three times with PBS (5 min each). Mounting medium with DAPI (4′,6-diamidino-2-phenylindole) (Vectashield H-1200, USA) was used for nuclear counterstain. Negative controls consisted of eliminating the primary antibodies for the procedure. The slides were examined under an epifluorescence microscope using a 20x and 40x objectives and photomicrographs were taken using the microscope imaging software (Leica Biosystems, Germany).

### 2.3. APN Injections in the OB

Previous reports had described that there was an important response to APN treatment within 72 to 96 h (Qi et al. [27]), and for this reason, APN response was measured at 72 h. We also performed dose-dependent assays starting from 500 ng and 1 and 2 *μ*g of APN and evaluated InsR protein expression at 72 h postinjection (*n* = 6). As there were no changes using different doses of APN (data not shown), we chose 1 *μ*g of APN for subsequent experiments as a mean parameter for the concentration of adiponectin. Then, two groups of animals (*n* = 6, each group) were anesthetized with a mix of ketamine-xylazine solution (1 ml/kg) and placed on a stereotaxic apparatus (KOPF Mod 5000, USA); then, stereotaxic injections were made by using a 10 *μ*l Hamilton syringe (model 701) coupled in manual microinjector with a velocity of 106 nl per revolution (Sutter Instrument USA). One group of animals received 1 *μ*l of unilateral injection of isotonic saline solution (SS) as control, and the treated group received an injection of APN (1 *μ*l at a dose of 1 *μ*g/*μ*l of APN oligomer (≈25.5 kDa), R&D Systems, 1065-AP-050, USA), directly into the OB according to the following coordinates, with reference to Bregma: (+7.5 mm anteroposterior, −1.0 mm lateral, and −4.0 mm dorsoventral) [28].

### 2.4. Protein Extraction and Western Blot Analysis

Western blot analysis of the total InsR content, phospho-InsR, and phospho-Akt was conducted in the OB homogenates. Both vehicle and APN-injected animals were sacrificed after 72 of the injection using a sodium pentobarbital overdose, and rapidly, their OB were dissected and then homogenized in RIPA lysis buffer (150 mM NaCl, 1% of sodium deoxycolate, 0.1% of sodium dodecyl sulfate, 50 mM Tris, and pH 8) supplemented with proteases (Roche, cat number 11697498001, USA) and phosphatases inhibitors (Roche, cat number 04906845001, USA). Proteins were quantified using a Micro BCA Protein Assay Kit (Pierce, cat number 23235, USA), and 60 *μ*g was separated by electrophoresis in 10% SDS-PAGE gels. Then, proteins were transferred to PVDF membranes (Merck Millipore, cat number ISEQ00010, USA), blocked with 5% nonfat dry milk diluted in 0.1% Tween-20 Tris-Buffered Saline (TBST) and incubated at 4°C overnight with rabbit polyclonal anti-p-AMPK (Thr 172) (cell signaling, cat number 2535, USA), rabbit polyclonal anti-total AMPK (Abcam, ab3760, USA), mouse monoclonal anti-*β*-InsR (Millipore, cat number 05-1104, USA), goat polyclonal anti-p-insulin R*β* (Tyr 1162/1163) (Santa Cruz Biotechnology, sc-25103, USA), or rabbit polyclonal anti-p-Akt 1/2/3 (Ser 473) (Santa Cruz Biotechnology, sc-7985-R, USA) antibodies diluted 1 : 1000. After incubation with the primary antibodies, membranes were incubated with anti-mouse (Santa Cruz Biotechnology, sc-2005), anti-goat (Santa Cruz Biotechnology, sc-2768, USA) or anti-rabbit (Santa Cruz Biotechnology, sc-2004, USA) horseradish peroxidase-coupled secondary antibodies diluted 1 : 10,000. Moreover, goat polyclonal anti-*β*-actin antibody (Santa Cruz Biotechnology sc-1616, USA) diluted 1 : 10,000 was used as loading control.

AdipoR1 and AdipoR2 protein content in the OB of intact rats (*n* = 6) was visualized with goat polyclonal anti-AdipoR1 (1 : 1000, Santa Cruz Biotechnology, sc-46748, USA) and rabbit polyclonal anti-AdipoR2 (1 : 1000, Santa Cruz Biotechnology, sc-99184, USA) following the same immunoblot protocol. Glyceraldehyde-3-phosphate dehydrogenase (GAPDH) (Sigma-Aldrich G8795, USA) was used instead of anti-*β*-actin as the loading control.

Immunoreactive bands were detected using chemiluminescence reactive (Millipore cat number WBLUF0500, USA) in higher performance film (Amersham cat number 28-9068-39, USA). The bands were analyzed in Image MCID Analysis Software (Interfocus, Imaging LTD, UK). The relative optical density of each band of different primary antibodies was normalized to its loading control.

### 2.5. RT-PCR Transcript Analysis

The total RNA was extracted from intact OB tissue (*n* = 5) using Trizol reagent (Invitrogen 15596-018, USA) following the manufacturer's instructions. In addition, gastrocnemius muscle and liver total RNA samples were used as positive controls for AdipoR1 and AdipoR2, respectively. The integrity of the total RNA was achieved by electrophoresis in 1% agarose gel prestained by GelRed dye (Biotium 41003, USA) observing the well-defined bands of 18S and 28S rRNA without smearing, and the concentration and purity were estimated spectrophotometrically at 206 and 280 nm ratio.

All RNA samples were treated with a DNA-free kit (Ambion AM1906, USA) to avoid genomic DNA contamination. Two micrograms of total RNA was used to synthesize the first strand of cDNA using the Rever Aid First Strand cDNA synthesis kit (Thermo Fisher Scientific K1622, USA) following the manufacturer's instructions. The cDNA was amplified by polymerase chain reaction by using Taq DNA polymerase (Thermo Fisher Scientific K0251, USA), 500 ng of cDNA OB sample, and the following gene-specific primers over 40 cycles in 10 *μ*l of total volume: AdipoR1, 5′CCACCATGCACTTTACTATC3′, forward and 5′ATGAGACTGGAACCATATGTC3′, reverse; AdipoR2, 5′TGACATCTGGTTTCACTTTC3′, forward and 5′TCATGAAACGAAATTCCTGC3′, reverse (KiCqStar primers, Sigma-Aldrich KSPQ12012, USA). Each sample was run in duplicate, and the PCR products were separated in a 2% agarose gels, stained with GelRed dye (Biotium 41003, USA) solution and visualized in a UV transilluminator.

### 2.6. Statistics

The values of relative optical units were reported in arbitrary units as the mean ± standard error (m ± SE). All dataset was examined to test the normality and then, two-tailed Student *t*-test was performed to compare control versus APN-treated group. Statistical tests were analyzed using Prism GraphPad statistics software, version 5.01 (GraphPad Software, USA). In all cases, *P* ≤ 0.05 was considered statistically significant.

## 3. Results

### 3.1. AdipoR1 and AdipoR2 Are Expressed in the OB in Rat Brain

**Figure 1:**
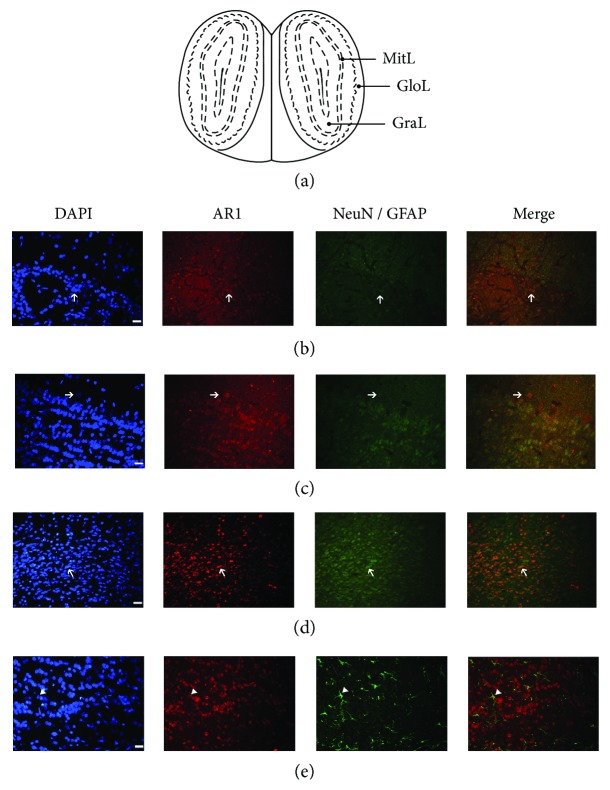
Presence and distribution of AdipoR1 in OB tissue from intact rats. Schematic representation of anatomical localization of the main OB layers (scheme modified from [28]) (a). As it is observed, AdipoR1 immunoreactivity is found in periglomerular (b), mitral (c), and granular cell layers (d). Double immunostaining shows that AdipoR1 colocalize with both neuronal marker (b and d, arrow) and astrocyte marker (e, arrowhead). Interestingly, mitral cells do not stain with NeuN marker. Nuclei were counterstained with DAPI to identify the different layers of OB. Scale bar corresponds to 20 *μ*m (40X). GloL: periglomerular cell layer; MitL: mitral cell layer; and GraL: granular cell layer.

**Figure 2:**
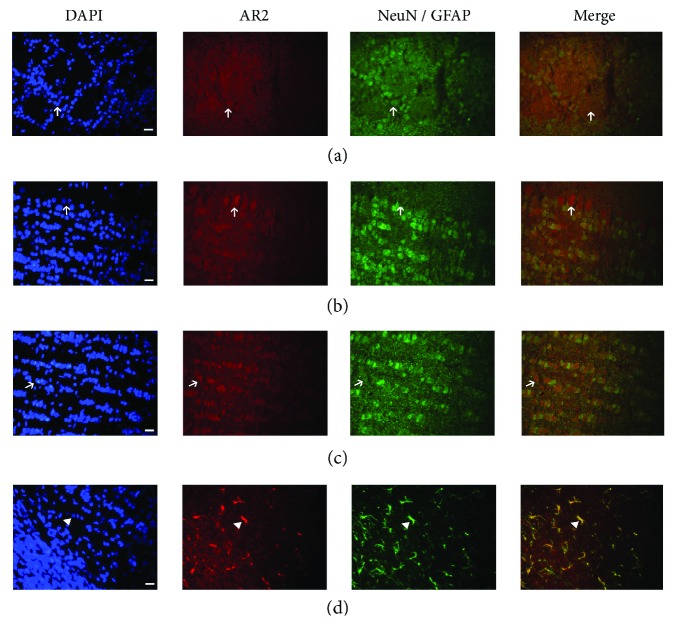
Distribution of AdipoR2 in OB tissue from intact rats. AdipoR2 immunoreactivity is also found in periglomerular (a), mitral (b), and granular cell layers (c); however, the mark of the immunoreactivity is less intense compared with AdipoR1 (Figure 1). Double immunostaining shows that AdipoR2 colocalize with both neuronal markers (a, b, and c, arrow) and had a strong mark with astrocyte marker (d, arrowhead). Note that mitral cells do not stain with NeuN marker. Nuclei were counterstained with DAPI to identify the different layers of OB. Scale bar corresponds to 20 *μ*m (40x).

To confirm the presence and distribution of AdipoR1 and AdipoR2 in the distinct layers of the olfactory bulb (Figure 1(a)), we performed immunofluorescence assays. As we can see, AdipoR1 was present in three main cellular groups of the OB such as periglomerular, mitral, and granular cell layers (Figures 1(b)–1(d), resp.); such immunoreactivity was detected in a diffuse and punctate pattern in the cells (arrow). Moreover, we also performed double immunofluorescence to characterize the cellular type that could be expressing the APN receptors. We observed that AdipoR1 is strongly colocalized with a neuronal marker (NeuN) (Figures 1(b) and 1(d)) and with astrocyte marker (GFAP) mainly in some parts of the astrocyte processes and some cases in the whole astrocyte (Figure 1(e), arrowhead). Interestingly, mitral cells do not stain with NeuN marker (Figure 1(c)). On the other hand, strong immunolabeling for AdipoR2 was also observed in the mitral cell layer (Figure 2(b)), whereas the expression was less intense in the periglomerular and granular layers (Figures 2(a) and 2(c)). Furthermore, double immunofluorescence assay showed colocalization with AdipoR2 and NeuN (Figures 2(a) and 2(c)) with the exception of mitral cells where there is no NeuN staining (Figure 2(b)) but strong colocalization within the whole astrocyte cells (Figure 2(d)). Interestingly, these findings were similar to those of Guillod-Maximin and colleagues where they showed that AdipoR1 is colocalized with NeuN marker and less extent with GFAP and AdipoR2 is strongly colocalized with both neuronal and glial markers in hypothalamic neurons [29].

**Figure 3:**
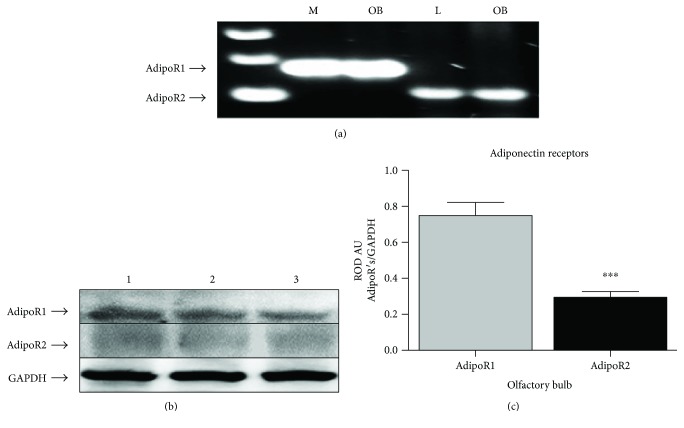
mRNA transcripts and protein content of both AdipoR1 and AdipoR2 in OB samples. RT-PCR analysis shows the presence of the mRNA transcripts corresponding to AdipoR1 (170 bp) and AdipoR2 (104 bp) in OB tissue. Skeletal muscle and liver were used as positive controls for AdipoR1 and AdipoR2, respectively (a). Furthermore, Western blot analysis shows protein content of adiponectin receptors in OB homogenates from intact rats (b), being AdipoR1 the receptor with major protein content compared with AdipoR2 (c). *n* = 6, ^∗∗∗^*p* < 0.001.

We assessed whether mRNA of both receptors was being expressed in OB tissue. By means of RT-PCR analysis, mRNA transcripts of both APN receptors were detected in the OB tissue and they correspond to those described in skeletal muscle (AdipoR1) and in the liver (AdipoR2) (Figure 3(a)). We also performed Western blot assays to examine protein content of APN receptors. As we can observe in Figure 3(b), both AdipoR1 and AdipoR2 protein contents are present in OB homogenates from intact rats and interestingly, there was a significative increase of the content of AdipoR1 compared with the AdipoR2 content (Figure 3(c)). These results demonstrated that OB is able to express both mRNA transcripts and the protein of APN receptors as in other brain regions (i.e., hypothalamus or hippocampus).

### 3.2. Differential Content of Total InsR, InsR, and Akt Phosphorylation after Injection of APN in OB

**Figure 4:**
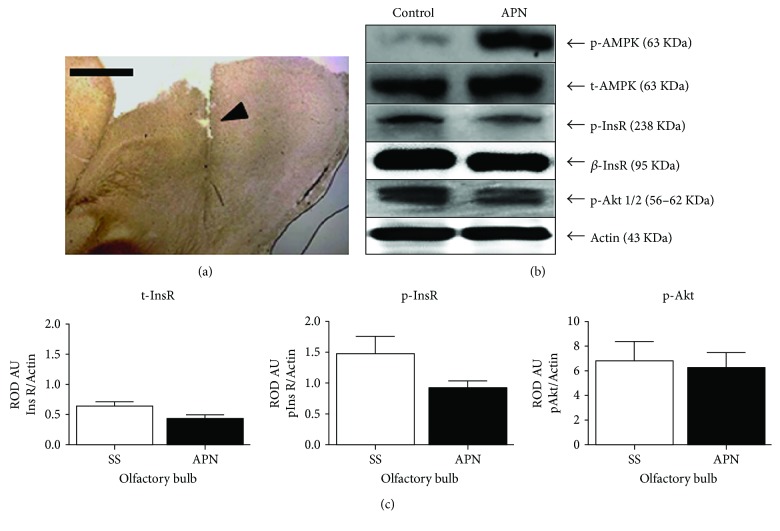
Effect of APN injection in OB on InsR protein content and insulin signaling components in OB homogenates. Photomicrograph shows the trajectory of the injection into OB (a). Representative images of immunoblotting show an increased in AMPK phosphorylated at Thr172 residue, a slight decrease of InsR content, a pronounced decrease of InsR phosphorylation, and slight reduction of phosphorylated Akt in OB homogenates 72 h after APN injection (b), respectively; however, none of the analyzed proteins had significative changes (c). Scale bar = 500 *μ*m.

In another series of experiments, we analyzed the possible effect of the injections of 1 *μ*g of APN in the right OB on the insulin signaling pathway by measuring the total content of InsR and InsR and Akt phosphorylation in contralateral OB.  Figure 4(a) shows the trajectory of the syringe in the OB to ensure the correct injection site (Figure 4(a)). First, we evaluated whether the adiponectin injection can activate its receptors and produce the transduction of the signaling pathway. Seventy-two hours after APN injection in the ipsilateral OB, injected animals showed an increase of AMPK phosphorylated at the residue Thr172 confirming that the APN injection produced a response activating its receptors (Figure 4(b)). Moreover, adiponectin injection produced a slight decrease in total InsR protein and a pronounced decrease in the phosphorylated-InsR compared with that of saline-injected animals; however, it was not statistically significant (Figures 4(b) and 4(c)). Moreover, the phosphorylation Akt kinase, a downstream component of insulin signaling pathway, was slightly reduced in protein content when compared with that of vehicle controls without being statistically significant (Figures 4(b) and 4(c)).

## 4. Discussion

Recent reports have described that peripheral adipokines and gut hormones may alter the perception and pleasantness of specific odors, presumably directly through their receptors in the olfactory system or indirectly through central interfaces between the regulation systems of olfaction, appetite control, memory, and motivation [30]. Moreover, a decreased in the sense of smell can lead to a significant impairment of quality of life, including taste disturbance and loss of pleasure from eating, which results in changes in weight and difficulty avoiding health risks, such as spoiled food [31].

As described in scientific literature, there is robust information about mechanisms where adiponectin (APN) is involved in metabolic regulation in peripheral tissues [32] and it has been proposed as a plasma marker of metabolic syndrome or type 2 diabetes mellitus [33]. APN exerts its function throughout the interaction with its receptors 1 and 2 (AdipoR1 and AdipoR2) which are widely expressed throughout CNS, including cortex, hypothalamus, and hippocampus [14]. Nevertheless, there were no reports describing that both gene and protein expression of AdipoR1 and AdipoR2 were present in the olfactory bulb of the rat brain even though the effect of APN in the responsiveness in olfactory system had been previously described [23]. In this regard, our results from immunoblot and immunofluorescence assays demonstrated the presence and a differential expression of AdipoR1 and AdipoR2 in different OB cell layers. The fact that we observed the differential expression of AdipoR1 and AdipoR2 in the periglomerular, mitral, and granular cell layers could be related with the roles that these three types of cells play in the olfactory system, that is, mitral cell process information from the olfactory epithelium while granular cells are modulators of this activity [34]. However, AdipoR1 expression was predominant in the OB when compared with AdipoR2, suggesting that APN signaling in the OB could be mainly mediated by AdipoR1 activation. Another puzzle is whether APN receptors have differential function according to their cellular type. As we have demonstrated, astrocytes and neuronal cells express both AdipoR1 and AdipoR2 receptors in OBs which agree with previous results where APN receptors are expressed in astrocyte and neuronal cell cultures [35] or in neurons and astrocytes in the arcuate and paraventricular hypothalamic nuclei [29]; however, very little is known about a specific function receptor for each cell type.

The presence of AdipoR1 in the olfactory epithelium of mice was attributed to a potential modulatory role of APN in the olfactory system [22]. Pretreatment with APN resulted in a higher response to an odorant stimulus in association with an increase in the neuronal activity of the periglomerular cells [23]. Furthermore, Guthoff and colleagues found a genetic variation in the promoter region of AdipoR1 that it is associated with decreased olfactory recognition in healthy human subjects [36]. As soon as the animals detect a food odor, an increase in the electrical activity of the mitral cells is observed [37]. According to these findings, we could hypothesize that whether an animal is in starving state, APN levels will increase in the periphery and it would cross to the blood-brain barrier and would bind to its receptors in different cells of the olfactory system (i.e., olfactory epithelium, and OBs) modulating the responsiveness to odors [23] and transmit the response to central brain structures, such as the hypothalamus to regulate food intake [29]. Recently, it has been observed that APN levels are increased in serum and cerebral spinal fluid (CSF) and the expression of the APN, and its receptor (AdipoR1 and AdipoR2) genes are upregulated in the liver and visceral adipose tissues in fasting condition [15, 38]. Nevertheless, experimental work is needed to prove such hypothesis.

On the other hand, glucose brain metabolism and olfactory function seem to be closely related; thus, the presence of InsR and AdipoRs throughout the brain suggests an interaction between APN and insulin activities in the central nervous system (CNS), particularly in the OB [39]. Unlike energetic and anabolic activities that insulin regulates in peripheral tissues, the putative roles of insulin in the CNS are related with feeding behavior regulation and energy expense, neurodevelopment, neuronal survival, learning, and memory and synaptic plasticity [26]. Even though the injection into the OB of recombinant APN protein had the physiological response when bound to its receptors (by increasing AMP phosphorylation), our results unexpectedly indicated that APN injection to the OB had little effect on due to the fact that there was slight decrease in the total InsR protein content, a pronounced reduction the phosphorylation of the InsR in the OB, and slight decrease of phosphorylation in the insulin downstream effector Akt using 1 *μ*g of APN. It is known that APN exists in several forms such as globular, trimers, hexamers, and high molecular weight and the binding of these forms to its receptors can lead to the stimulation of several proteins such as AMPK, p38-MAPK, JNK, and PPAR*α* [14]. In the present work, we injected commercial APN protein (presumably monomers). However, we cannot assume or discard the formation or trimers, hexamer, or HMW oligomers [15] to explain, in part, the poor response of the APN injection in the content of insR or its phosphorylation. Nevertheless, we believe that adiponectin injection does not have a role in the production or activation of insulin signaling pathway at least in olfactory bulb region. Conversely, APN injection into hippocampus increases the total content of InsR, the phosphorylation of the InsR, and the phosphorylation of Akt (data not shown). According to these findings, we hypothesize that APN could influence the InsR expression and downstream signaling in a tissue-specific manner in the brain depending on the dose or the time after APN injection. In this regard, it has been shown that insulin is a strong modulator in the OB and an increase in insulin levels due to food intake could modulate its activity [40]. Thus, the link between the olfactory system and energy balance should not be unexpected given that the perception of odorants drives to food intake and selection [30]. Moreover, APN functions as an insulin sensitizer and the adaptor protein of AdipoRs, the phospho-tyrosine interacting with PH domain and a leucine zipper 1 (APPL1) mediates the activation of insulin signaling which could have an important role in the crosstalk between adiponectin/insulin signaling pathways in target tissues [14, 41].

## 5. Conclusions

In summary, we demonstrated that AdipoR1 and AdipoR2 are expressed in the main cell layers of the OB having differential distribution. Also, the dose of APN used in the stereotaxic injection activates its receptors but it does not seem to play an important role in the regulation of InsR expression, InsR phosphorylation, and Akt phosphorylation in the OB. Nevertheless, we hypothesize that APN could modulate the insulin pathway in other brain regions, suggesting that it is able to regulate a variety of cellular processes, including olfactory functions at different levels. A better understanding of how APN is linked to insulin signaling in the CNS is necessary because it is important to know whether this relationship could serve as a possible marker of brain activities and reveal potential therapeutic targets.
